# A Novel Biomass-Based Catalyst Composite Using Waste Chicken Eggshells and Avocado Seeds for Biolubricant Production: Synthesis Route, Catalytic Property Characterization, and Performance

**DOI:** 10.3390/molecules30214280

**Published:** 2025-11-03

**Authors:** Juan Esteban Foronda-Quiroz, Hilda Elizabeth Reynel-Ávila, Luiz Pereira-Ramos, Adrián Bonilla-Petriciolet

**Affiliations:** 1 Tecnológico Nacional de México—Instituto Tecnológico de Aguascalientes, Aguascalientes 20256, Mexico; esteban.421@hotmail.com (J.E.F.-Q.); petriciolet@hotmail.com (A.B.-P.); 2 Graduate Program in Chemical Engineering, Federal University of Parana, R. Francisco H. dos Santos, Curitiba 81531-990, PR, Brazil; luiz.ramos@ufpr.br; 3 Secretaría de Ciencia, Humanidades, Tecnología e Innovación, Av. Insurgentes 1582 Sur, Mexico City 03940, Mexico

**Keywords:** bioproducts, biomass waste, heterogeneous catalyst, trimethylolpropane fatty acid triester

## Abstract

This study introduces the preparation and tailoring of the catalytic properties of a novel biomass-based composite to produce a sustainable biolubricant, trimethylolpropane fatty acid triester (TFATE), via the transesterification of fatty acid methyl esters (FAMEs). This novel catalyst was prepared from avocado seed and chicken eggshell residues using a Taguchi experimental design to determine the best synthesis conditions. The variables tested in the catalyst preparation included CaO impregnation time and temperature, mass ratio of CaO/char, and activation temperature. The transesterification conditions to obtain TFATE were analyzed using the best eggshell-/char-based catalyst, and the reaction kinetics were measured at 120 and 150 °C. The results showed an endothermic reactive system with calculated kinetic rate constants of 7.45 × 10^−3^–10.31 × 10^−3^ L/mmol·min, and an activation energy of 15 kJ/mol. This new catalyst achieved 90% TFATE formation under optimized reaction conditions. Reuse tests indicated that catalyst deactivation occurred due to active-site poisoning, despite very low calcium leaching. Catalyst characterization confirmed the relevance of the crystalline structure and CaO loading on the avocado char surface to obtain the best catalytic properties, while ^1^H nuclear magnetic resonance analysis proved TFATE formation. This low-cost catalyst can be an alternative for enhancing sustainable biolubricant production with the aim of replacing petrochemical-based counterparts.

## 1. Introduction

Lubricants are multifunctional materials that can be utilized to reduce friction, transmit energy, prevent corrosion, dissipate heat, and act as sealants [1,2]. For decades, the petroleum industry has consolidated lubricant production via the introduction of additives and improved chemical techniques for their synthesis, establishing petroleum-derived oils as universal lubricants for a wide range of industrial and commercial applications [3,4]. Worldwide production of lubricating grease is estimated at ~1.0 million tons annually, with a market value of USD 3.50 billion by 2021 [5]. The global demand for grease is largely dominated by mineral-oil-based products, accounting for 90% of the total market [5]. However, the life cycle of mineral oil lubricants can negatively affect the environment, posing potential risks to public health and ecosystems [6]. It is estimated that over 50% of lubricants worldwide are managed inadequately, leading to spills into water bodies and total-loss applications such as chainsaw oils and two-stroke engine lubricants [7,8]. The overexploitation of global petroleum reserves, continuous fluctuations in crude oil prices, and environmental protection have driven the exploration of environmentally friendly and green alternatives to serve in place of conventional petroleum-based lubricants [9,10]. It has been estimated that 90% of the developed lubricants can be replaced by bio-based options [11]. Consequently, there is a need to develop low-cost synthesis routes to obtain biodegradable products that can substitute their petroleum-based counterparts [4,5,12,13,14]. Biolubricants offer significant advantages over mineral-oil-based lubricants because they can be produced from environmentally friendly raw materials that promote sustainable economic growth in less developed areas, and their use can generate biodegradable compounds; in addition, the adoption of sustainable processes for their production is aligned with the principles of the circular economy [8]. Despite these advantages, it has been estimated that the production and commercialization of synthetic esters comprise 9% of the total market, whereas biodegradable-oil-based greases represent only 1% [5].

**Figure 1:**
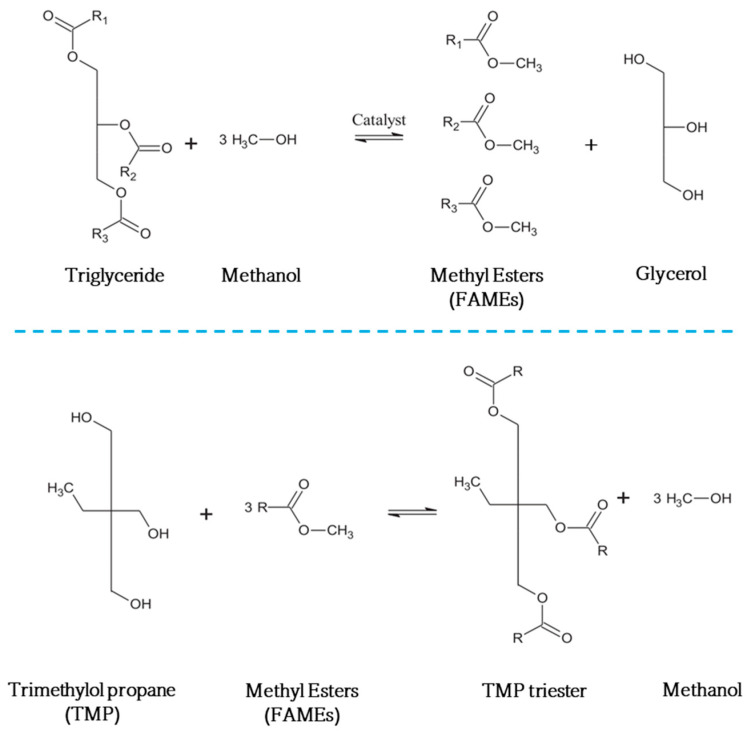
Synthesis of polyolesters from vegetable oils using trimethylolpropane (TMP). R_1_, R_2_, and R_3_ are different alkyl groups.

A potentially promising source of biolubricants is vegetable oils obtained from plants, such as sunflower, soybean, rapeseed, coconut, palm, karanja, and jatropha [15]. Cooking oils and microalgae have also been investigated for use in biolubricant production [3]. Nevertheless, the low oxidative stability and high melting point of these oils limit their application as lubricants and, consequently, chemical modifications (e.g., hydrogenation, epoxidation, and transesterification) have been proposed to improve the properties of vegetable oils [16,17]. Transesterification is the conventional and preferred method for producing polyolesters oils (biolubricants) from vegetable oils and animal fats [18]. This reaction is generally carried out using methyl esters of fatty acids (i.e., FAMEs already synthesized from a previous reaction) in the presence of a branched long-chain alcohol, such as trimethylolpropane (TMP) or ethylene glycol, where a catalyst is often used (see Figure 1) [2,19]. The production of polyolesters via transesterification removes the hydrogen atom at the β-position of glyceride, which in turn increases the biolubricant’s stability [20]. To date, several studies have explored the preparation of different types of biolubricants by analyzing the type of raw material, alcohol, or techniques used for their production, proving that biolubricants with diverse characteristics can be prepared; however, the costs associated with both raw materials and catalysts used in the production of biolubricants have driven the search for better approaches.

Heterogeneous biomass-derived catalysts are alternatives to conventional catalysts for reducing the production costs of biolubricants and improving their carbon footprints [21]. These catalysts offer several advantages compared to their counterparts. They can be prepared from naturally abundant and low-cost resources and stand out for their low environmental impact because they are neither corrosive nor toxic [22]. Specifically, calcium oxide (CaO) is a competitive heterogeneous catalyst with high alkalinity and catalytic activity that can be derived from waste such as shells from chicken eggs, ostrich eggs, snails, oysters, and crabs [23,24]. For example, Ghafar et al. [21] obtained CaO from waste cockle shells and used it as a heterogeneous catalyst to produce a biolubricant from waste cooking oil; TMP triester biolubricant with 97% conversion was obtained in that study. Iyya et al. [25] prepared CaO from eggshells in a muffle furnace at 800 °C and activated the oxide using H_3_PO_4_. This catalyst was used in the esterification of neem seed oil with TMP to obtain a biolubricant. Unfortunately, the biolubricant yield was not reported, and the effect of the reaction conditions was not analyzed by the authors.

Despite its benefits, CaO may exhibit instability and partial solubility in alcohols, leading to leaching and sensitivity to moisture and CO_2_, which can result in catalyst deactivation [24,26,27]. To address these challenges and enhance the catalytic performance, CaO can be combined with other metal oxides or supported on materials such as biochar and silica [24,26,27,28]. Biochar is particularly promising as a catalyst support because of its exceptional properties, including thermal, mechanical, and structural stability, cost-effectiveness, environmental friendliness, biodegradability, high porosity, and ease of functionalization [27]. Some studies have proposed the synthesis of catalysts that incorporate CaO onto the biochar surface for biodiesel production [28,29,30,31]. Wang et al. [28] studied the anchoring of CaO onto peat char for biodiesel production from palm oil and achieved 93% yield. The best reaction conditions were identified, and the catalyst was reused for 10 cycles with a final biodiesel yield of 82%. This material exhibited high catalyst stability, which was attributed to the Ca-O-Si bonds formed on the char surface. In another study, biochar prepared from municipal sludge was impregnated with CaO and used for the transesterification of palm oil to obtain a biodiesel yield of 94%, which decreased to 85% after ten cycles [29]. Das et al. [30] synthesized a biochar from switchgrass that was subsequently functionalized with CaO from eggshells. This catalyst was used in biodiesel production from algal lipids, obtaining a 99% yield. After 10 reuse cycles, this catalyst achieved 77% biodiesel yield. However, to the best of the authors’ knowledge, studies on the performance of metal oxides supported on char for biolubricant production have not been extensively addressed; few studies have analyzed this topic. For instance, Gao et al. [20] synthesized a catalyst consisting of K_2_CO_3_/activated carbon. Commercial activated carbon was impregnated with K_2_CO_3_ at different concentrations (10–40% by mass), and then activated at 500 °C under N_2_ flow for 3 h. Catalyst performance was tested in the transesterification of methyl oleate with TMP using microwave irradiation. The reaction conditions were optimized to obtain 94% of biolubricant trimethylolpropane fatty acid triester (TFATE) formation, and the catalyst was reused 5 times.

The present study aimed to assess biolubricant TFATE production using a novel catalyst composite prepared from biomass residues, namely, avocado seeds and chicken eggshells. Avocado seeds were pyrolyzed to obtain biochar that was used as a support for CaO loading, which was obtained from chicken eggshells. The best catalyst preparation route was selected from a Taguchi experimental design, and the effects of reaction conditions on TFATE formation were evaluated, including the assessment of eggshell-/char-based catalyst reuse tests. The experimental transesterification data were modeled to investigate the biolubricant reaction parameters, and detailed characterization of the catalyst samples and reaction products was performed. In summary, a new heterogeneous catalyst for sustainable biolubricant production is introduced in this manuscript, which can be applied to support the low-cost transition of petrochemical-based lubricants to bio-based alternatives.

## 2. Results and Discussion

### 2.1. Preparation and Performance Tailoring of Eggshell-/Char-Based Catalyst to Obtain TFATE

The catalytic performance of nine eggshell-/char-based catalysts in the production of TFATE is shown in Table 1. Levels of TFATE formation ranged from 47 to 70%, with the best performance observed for non-optimized catalyst C2, while catalyst C9 showed the worst catalytic properties for the tested reactive system. This difference was attributed to the variation in the catalyst synthesis conditions.

**Figure 2:**
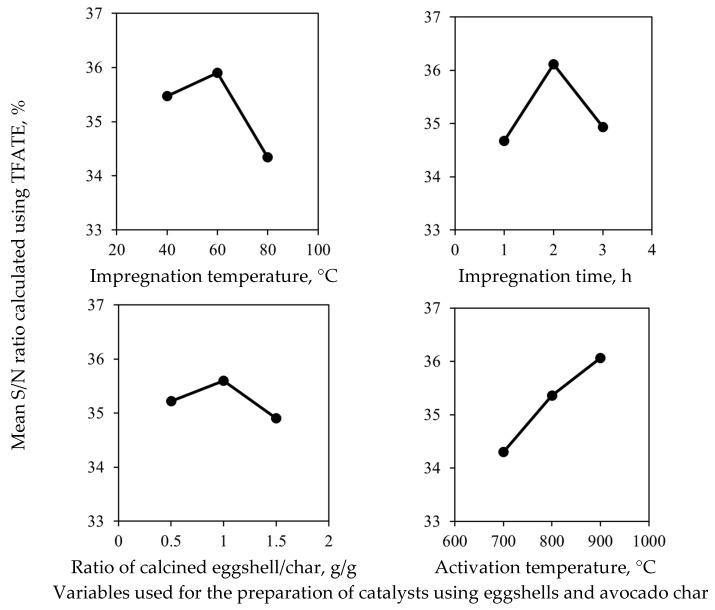
Results for Signal/Noise ratio analysis for the synthesis of heterogeneous catalysts from eggshell and avocado seed to produce lubricant TFATE via transesterification.

In particular, the catalysts with the highest levels of TFATE production were samples C2, C4, and C8, where two of them were activated at 900 °C. In contrast, the catalysts with the lowest levels of TFATE formation were samples C1, C7, and C9, which were activated at 700–800 °C. Consequently, these results suggest that the activation temperature was a critical factor to favor TFATE formation, which could be attributed to the fact that calcium oxide is formed at ≥800 °C [32]. Overall, the relevance of tested synthesis variables to tailor the catalyst performance was as follows: activation temperature > impregnation temperature > impregnation time >> eggshell/char ratio, see Figure 2 and Table 2.

The Signal/Noise (S/N) ratio analysis confirmed that the activation temperature had a statistically significant effect on the catalytic properties of eggshell-/carbon-based materials used for obtaining TFATE, where the catalytic properties increased with the activation temperature, see Figure 2 and Table 2.

This phenomenon can be explained by the formation of CaO at high temperatures, which gives the eggshell/char composite its catalytic activity. Previous transesterification studies have demonstrated the high catalytic efficiency of CaO, indicating that the cost of this material is lower than that of other catalysts [10]. For instance, Vanthana et al. [32] reported that high temperatures (>800 °C) for long periods (>3 h), or 900 °C for shorter periods (1 h), favor the formation of CaO.

Therefore, it can be concluded that the activation temperature was the main variable for the synthesis of this eggshell/char composite because of the conversion of CaO on the catalyst surface. On the other hand, the statistical analysis also showed that the variables involved in the mixing of calcined eggshells and avocado char in the aqueous solution also affected the catalytic properties of the eggshell-/char-based material to obtain the biolubricant, although their trend was nonlinear, see Figure 2. These two variables affect the distribution of calcium particles on the char surface (i.e., the catalyst support).

Therefore, their best values were identified to favor the effective diffusion of the species and an improved distribution of CaO on the char that worked as a catalytic support. In contrast, the eggshell/char ratio did not significantly affect biolubricant yield. Based on these facts, the best catalyst synthesis conditions to maximize TFATE formation were defined as follows: mixing of calcined eggshells and char in aqueous solution was performed at 60 °C for 2 h with an eggshell/char ratio of 1/1 (g/g), and the eggshell-/char-based composite was activated at 900 °C for 1 h.

### 2.2. Kinetics for TFATE Production Using the Best Eggshell/Char Catalyst, Reaction Condition Analysis, and Catalyst Reuse

**Figure 3:**
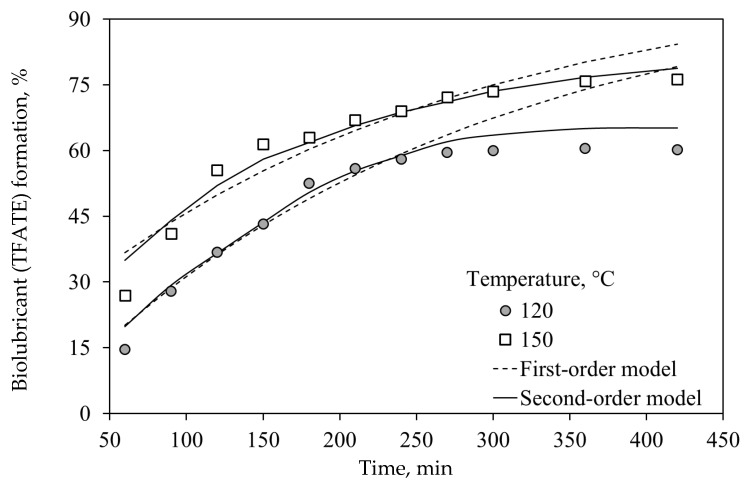
Transesterification kinetics of FAMEs to produce the biolubricant (TFATE) at different temperatures using the eggshell-/char-based catalyst. Reaction conditions: 5% (by mass) catalyst load, 3/1 molar ratio of FAME/TMP, and 0.4 atm.

TFATE reaction kinetics using the best eggshell/char catalyst at 120 and 150 °C are shown in Figure 3. No notable changes were observed in TFATE formation after 240 min of reaction at 120 °C, whereas the equilibrium for this reactive system was achieved at 300 min at 150 °C. These results suggest that transesterification requires long reaction times to achieve the highest levels of biolubricant formation. For comparison, the FAME reaction can achieve equilibrium in 60 min at 50–70 °C using heterogeneous biomass-based catalysts [33]. This trend could be due to the use of polyols (e.g., trimethylolpropane), which are solid at room temperature and make transesterification more complex compared to the use of other alcohols. Therefore, this reactive system requires extreme reaction conditions: first, for the alcohol to be in liquid form, and then to start the reaction [8]. Similar findings were discussed by Maximo et al. [34], who concluded that the use of branched alcohols had a predominant effect on the reaction rate via steric hindrance, thus slowing its rate.

**Figure 4:**
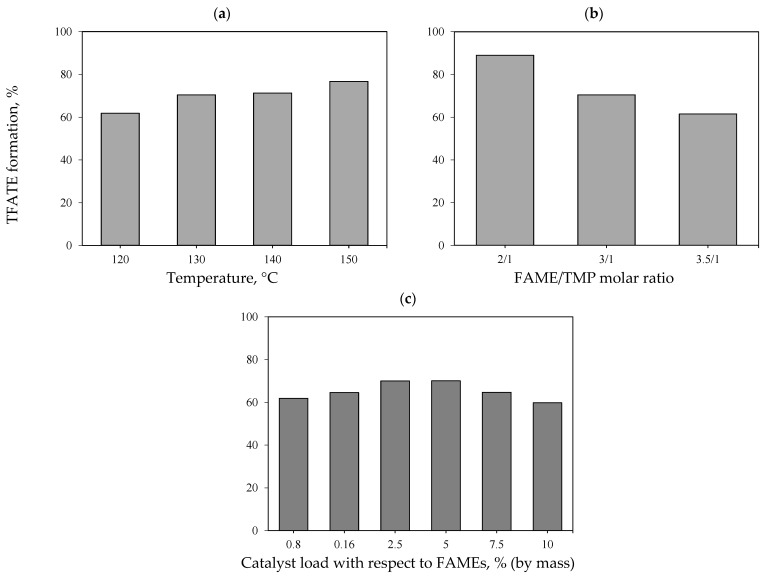
Impact of reaction conditions on biolubricant TFATE formation: (**a**) transesterification temperature, (**b**) FAME/TMP molar ratio, and (**c**) catalyst load. Nominal values of reaction conditions: 130 °C, 400 mbar, FAME/TMP molar ratio of 3/1, and catalyst load of 5% (by mass).

It was also determined that an increase in the reaction temperature had a direct effect on the transesterification rate and biolubricant yield, see Figure 4a. In particular, the transesterification to produce TFATE using eggshell-/char-based catalyst was endothermic, which agrees with previous studies [35]. Because an endothermic reaction requires heat, the reaction temperature increase promotes a higher conversion of reactants into products. An increase of 27% in TFATE production was observed when comparing the reaction at 150 °C (TFATE yield: 76%) with that at 120 °C (TFATE yield: 61%). The calculated transesterification rate constants increased when the reaction temperature changed from 120 to 150 °C (see Table 3), confirming its favorable impact on TFATE formation.

Specifically, the first-order rate constants were 3.74 × 10^−3^ and 3.88 × 10^−3^ min^−1^, while the second-order rate constants were 7.39 × 10^−3^ and 10.25 × 10^−3^ L/mmol·min at 120 and 150 °C, respectively. The second-order kinetic model was the best option to describe the experimental transesterification data with R^2^ = 0.97–0.98. Consequently, the calculated activation energy of this reactive system was 15 kJ/mol. Note that activation energy values of <30 kJ/mol suggest that the reaction rate may be restricted by diffusion or mass transfer processes [35,36,37], indicating the need to overcome a barrier to achieve the mixing of reagents for starting biolubricant formation.

Similar findings were documented by Menkiti et al. [36] in TFATE synthesis using jatropha oil, TMP, and calcium hydroxide as a catalyst. These authors calculated an activation energy of 14 kJ/mol via a second-order kinetic model. Encinar et al. [37] also reported analogous results in TFATE production using canola oil, TMP, and sodium methoxide (catalyst) with an activation energy of 7 kJ/mol via second-order reaction kinetics.

The effect of the FAME/TMP molar ratio on biolubricant yield is shown in Figure 4b, where a maximum TFATE formation of 90% was obtained using a FAME/TMP molar ratio of 2/1. This operating variable exhibited an inverse correlation; that is, as the proportion of FAME increased compared to that of TMP, the biolubricant yield decreased. This phenomenon was associated with the intrinsic reaction characteristics, which have a 3/1 stoichiometry of FAME/TMP [37], meaning that alcohol reduction affected the complete conversion to biolubricant due to stoichiometric limitations. This result agreed with the study performed by Ghafar et al. [21] on the production of a biolubricant from waste cooking oil, TMP, and CaO obtained from the calcination of clam shells. These authors reported that biolubricant formation decreased from 97 to 89% when the FAME/TMP molar ratio was increased from 3/1 to 4/1. These results prove that excess FAME content shifts the reaction equilibrium back towards reactant formation. This could also result in TFATE reduction due to catalyst dilution caused by an excessive amount of FAMEs. Note that Encinar et al. [8] determined that the excessive use of alcohol favors the reaction towards product formation. However, it was found that FAME/TMP ratios < 1/1 generated operational difficulties for removing excess alcohol, which was absorbed into the biolubricant, thus complicating its characterization analysis and quantification.

On the other hand, the impact of the catalyst loading on the reaction yield is shown in Figure 4c. A clear trend of increasing biolubricant yield as the catalyst concentration increased was observed within the range of 0.8 to 2.5% (by mass). No significant changes were observed after reaching a catalyst load of 2.5% (by mass). In fact, the biolubricant yield decreased when the catalyst concentration exceeded 5% (by mass). High catalyst concentrations hindered effective mixing between the reactants, product, and catalyst itself, which favored the reversible reaction [38]. For illustrative purposes, Table 4 shows a comparison of TFATE formation using various catalysts and reagents under different operating conditions. Levels of TFATE formation reported in the literature range from 88 to 97%. These results indicate that the novel eggshell/char composite reported in this manuscript is a low-cost, competitive catalyst.

The calcium leaching test using methanol indicated that 0.056 mg of Ca was released from this catalyst per gram of methanol, which represents only 0.018% of the total Ca content of the tested catalyst. Table 5 shows the levels of TFATE formation during five reaction cycles and the corresponding calcium content found in the biolubricant. These results showed that catalyst deactivation resulted in 48% TFATE formation in the fifth reaction cycle. It is important to note that the fresh catalyst contained a calcium content of 334.8 mg/g, while the calcium leached into the biolubricant ranged from 0.038 to 0.092 mg/g during the five reaction cycles, with a total leached Ca amount of 0.332 mg/g.

Therefore, only 0.1% of the total calcium content of the catalyst was leached during these experiments. Because low calcium leaching into TFATE occurred, it can be speculated that catalyst deactivation was due to the presence of reagents and reaction products that blocked the catalyst active sites [39].

Similar catalyst deactivation results were reported by Etim et al. [40]. These preliminary findings suggest that a better regeneration catalyst route should be proposed to recover the catalytic properties of eggshell-/char-based composite.

### 2.3. Characterization of Avocado Biomass, Catalysts, and Reaction Products

The biomass extraction results indicated that ~20% (by mass) of avocado seeds was extracted using water. The washing liquid from this agricultural waste showed a carbohydrate content of 4.99 mg/mL, while the structural carbohydrate and lignin contents were as follows: 79.8% cellulose, 2.6% hemicellulose, 2.1% acid-soluble lignin, and 10.1% acid-insoluble lignin. The remaining percentage is associated with lipids, proteins, and polyphenolic compounds [26]. This composition analysis confirmed that avocado seeds can be used in the catalyst preparation [41], and also suggests their potential for other bioenergy applications, including biogas production via anaerobic digestion as well as biofuel production such as biodiesel and bioethanol [26]. Thermogravimetric analysis (TGA) results of the avocado-seed sample are shown in Figure S1, where a mass decrease of 52.9% at 280–390 °C was observed. This thermal behavior can be explained by the fact that the degradation temperature of cellulose, which is the main component of avocado seeds, is close to this temperature range. The elemental analysis of avocado seeds indicated that their composition included oxygen (48.54%), carbon (44.25%), and hydrogen (6.59%), besides trace elements such as nitrogen, sulfur, and silicon, see Table 6. This elemental content was consistent with reported values for other lignocellulosic biomass samples [42,43].

**Figure 5:**
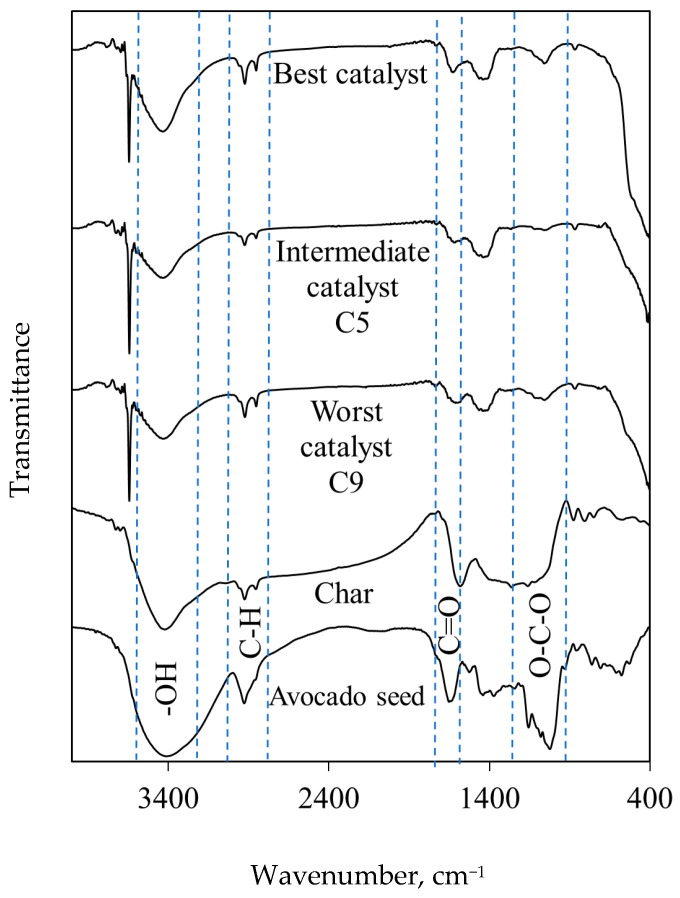
FTIR spectra of avocado seed, char and eggshell/char-based catalyst used in TFATE production.

**Figure 6:**
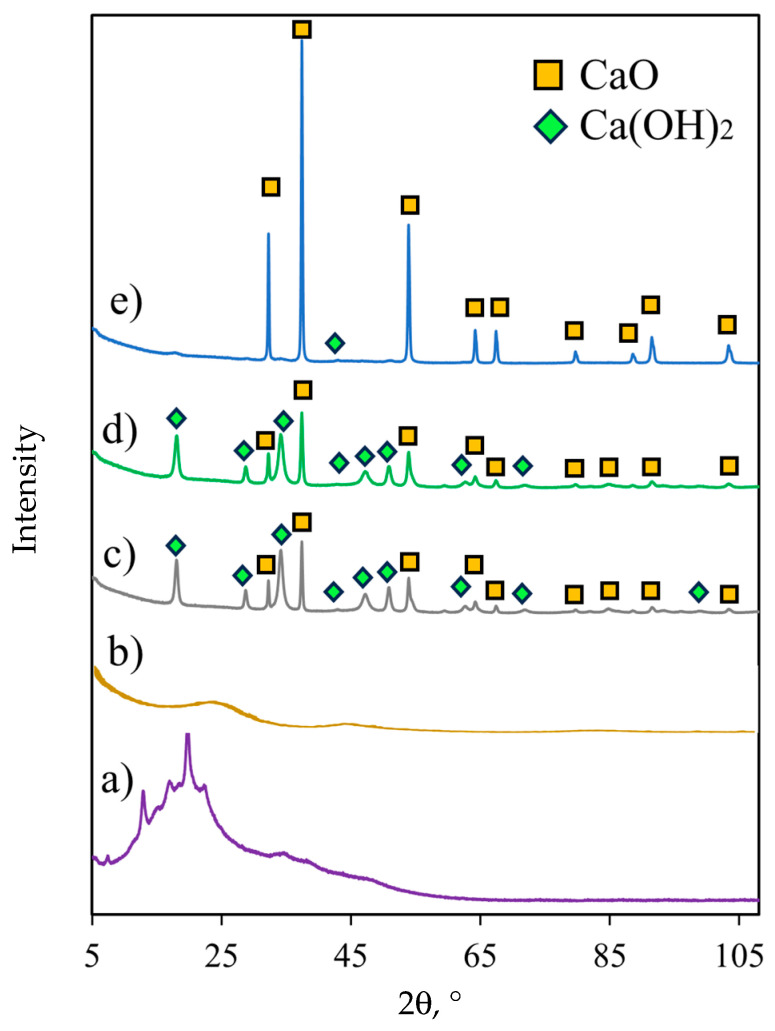
XRD diffractograms of (a) avocado seed, (b) char, (c) the worst catalyst C9, (d) the intermediate catalyst C5, and (e) the best catalyst.

The Fourier-transform infrared spectroscopy (FTIR) spectrum of the avocado seeds is displayed in Figure 5, where the absorption bands of typical functional groups found in the lignocellulosic biomass were observed [43]. The wide absorption band at ~3400 cm^−1^ was ascribed to OH groups present in alcohols [44,45], while the absorption bands of C-H aliphatic vibrations were located at 2970–2850 cm^−1^ [43]. The absorption bands between 1660 and 1510 cm^−1^ were related to OH group vibrations (e.g., aldehydes and carboxylic acids), the C=O of the carbonyl group, cellulose cyclic C-C structures, and lignin aromatic C=C groups. The absorption bands identified at 1350–1060, ~1015, and ~910 cm^−1^ correspond to C-O vibrations in primary, secondary, and tertiary alcohols, as well as the glycosidic bonds contained in cellulose and hemicellulose [42,43,46,47]. The absorption bands of vibrations linked to cyclic structures were found between 700 and 600 cm^−1^ [48,49]. The X-ray diffractogram of the avocado-seed sample confirmed the presence of a semi-crystalline structure, see Figure 6. The main diffraction peaks were located at 12.97, 17.01, 22.45, and 34.0 °2Ɵ, which were related to the crystalline phases of cellulose [42,50]. Note that the wide halo centered at 20 °2Ɵ suggests the presence of amorphous fractions of lignin.

The elemental analysis of the avocado-based char sample is shown in Table 6. A significant increase in the carbon content in this sample was observed after the biomass pyrolysis (i.e., from 44 to 84%). This behavior is usually observed in lignocellulosic biomass pyrolysis, mainly due to the devolatilization of aliphatic functional groups and the aromatization/carbonization of char [51,52].

FTIR spectrum of avocado char shows the loss, decrease, and shrinkage in various absorption bands that were identified during the biomass analysis, see Figure 5. These changes were especially associated with the absorption bands of OH and C-O vibrations located at ~ 3400, 1400, and 1150–1000 cm^−1^, which were caused by material structural changes because of the pyrolysis process. The thermal biomass decomposition results in the material structure being reorganized and recombined via the breakage of glycosidic linkages, compound volatilization, aromatization, condensation, and other reactions [42,51,52,53].

On the other hand, the FTIR spectra of the catalyst samples contained a sharp absorption band at ~3640 cm^−1^ associated with the hydroxyl group of Ca(OH)_2_ [26,54], and a broad absorption band at ~3420 cm^−1^ attributed to the OH vibration of functional groups present on char such as phenols and alcohols of cellulose, hemicellulose, and lignin [54]. For illustration, the FTIR spectra of the best catalyst and samples C9 (with the worst performance) and C5 (with intermediate performance) are shown in Figure 5. In the wavenumber region of 1650–1420 cm^−1^, the absorption band of the vibration COO- of the Ca–carboxylate coordination type was observed [54]. The absorption band located at ~1056 cm^−1^ was related to the C-O stretching vibration of alcohols [26,54]. It is important to remark that the intensity of absorption bands displayed in these spectra was relatively higher for the best catalyst, except for the absorption band at ~3640 cm^−1^ related to Ca(OH)_2_. This finding may be attributed to the strong interactions between the functional groups on char and calcium species [54,55].

The X-ray diffraction (XRD) patterns of selected catalyst samples are shown in Figure 6. The diffractogram of avocado char displays two broad peaks at 25 and 45 °2Ɵ, associated with both the graphitic structure and amorphous carbon [56]. After the impregnation of this char with the calcium solution, the appearance of a crystalline structure was evident. The peaks corresponding to calcium hydroxide and calcium oxide were identified in the catalyst samples at different proportions, according to the activation temperature. The PDF-2 database indicated that the peaks at 18, 28, 34, 47, 50, 62, 71, and 84 °2Ɵ were related to calcium hydroxide (ICDD: 00-044-1481). On the other hand, the characteristic peaks of calcium oxide (ICDD: 00-043-1001) appeared at 32, 37, 53, 64, 67, 79, 88, 91, and 103 °2Ɵ [57]. A semi-quantitative analysis showed that the best catalyst sample contained 99% of CaO and 1% of Ca(OH)_2_, while the worst catalyst (sample C9) contained 45% of CaO and 55% of Ca(OH)_2_. As stated, the activation temperature of eggshell/char composite was paramount to confer higher catalytic properties [32]. The XRD results indicate that the crystalline structure of the catalyst is associated with its catalytic activity.

Table 7 provides the elemental composition of selected catalyst samples, demonstrating that the superficial Ca content (42.8%) of the best catalyst was higher than that of the worst catalyst (24.9%). Other elements such as Al, Mg, Si, S, and Cu were found in amounts lower than 1%. This result reaffirmed that calcium is fundamental for the catalytic properties of eggshell-/char-based composites, with a direct correlation to TFATE formation. On the other hand, the BET surface areas of the tested catalysts ranged from 50 to 70 m^2^/g. The analysis shows that this parameter did not play a relevant role in determining catalyst performance.

**Figure 7:**
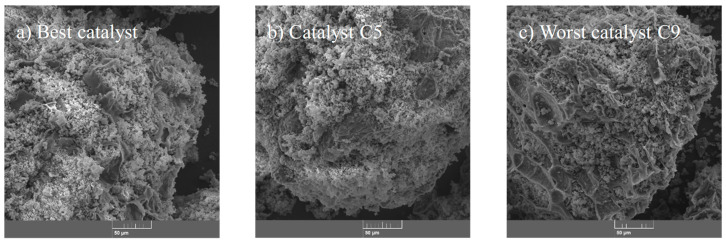
SEM images of selected samples of eggshell-/char-based catalysts used for biolubricant (TFATE) formation. SEM magnification: 1000×.

SEM micrographs of different catalyst samples are shown in Figure 7. All samples had a compact, eroded, and irregular shape. It is worth noting that the best catalyst presented a more saturated surface with Ca particles with homogeneous distribution, while the intermediate and worst catalyst samples showed a smooth surface with cavities of char exposed, and fewer Ca particles were observed according to the EDS mapping results (Figure S2). Overall, Ca concentration in the tested samples followed the tendency: best catalyst > intermediate catalyst > worst catalyst.

Table 8 shows the fatty acid composition of FAMEs, where oleic acid was the main compound with 90.84%, followed by palmitic acid with 4.66%. Herein, it is convenient to recall that the profile of fatty acids present in oils is a critical factor that influences the final properties of the biolubricant. Oleic acid (C18:1) contains one unsaturation in its chain, which is important for biolubricant oxidative stability because it is less susceptible to oxidation compared to linoleic (C18:2) and linolenic (C18:3) acids, thus contributing to the stability of the final products obtained via transesterification [37].

**Figure 8:**
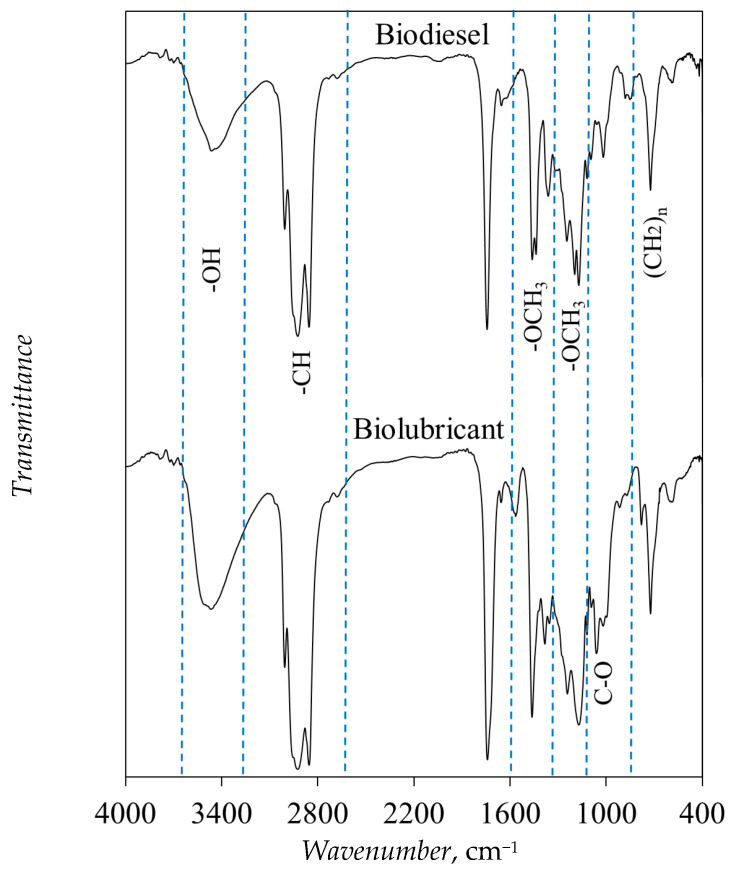
FTIR spectra of biodiesel (FAMEs) and biolubricant (TFATE) obtained using eggshell-/char-based catalyst.

FTIR spectra of the biodiesel (FAME) and biolubricant (TFATE) are displayed in Figure 8. The FAME spectrum shows the existence of an absorption band of hydroxyl group (-OH) in the esterified oils [58]. The characteristic absorption bands in the region of 3000–2850 cm^−1^ corresponded to the stretching vibrations of the C-H bond [58]. A distinctive absorption band located at ~1740 cm^−1^, related to the stretching vibration of the C=O double bond in the ester, was observed [59].

The absorption band of the bending vibrations of the C-H ester bond was identified at ~1461 cm^−1^ [58]. Notable absorption bands were identified at ~1170 and ~1118 cm^−1^, which were associated with the antisymmetric, asymmetric, and symmetric C-O-C bond, indicating the existence of fatty acid chains [10,58,59]. The absorption bands attributable to the alkyl chain were observed at ~1363 and ~723 cm^−1^ [58,60]. Two sharp bands at ~1437 and ~1196 cm^−1^ were associated with the bending and stretching vibrations of -OCH_3_ of esters [58]. In the TFATE spectrum, the appearance of a new absorption band at ~1056 cm^−1^ confirmed the formation of the C-O bond of TFATE [61]. Also, the disappearance of the absorption bands located at ~1437 and ~1196 cm^−1^, related to the methoxy group, implied the successful conversion of FAMEs into TFATE [58]. Similar results have been reported by Shrivastava et al. [60].

**Figure 9:**
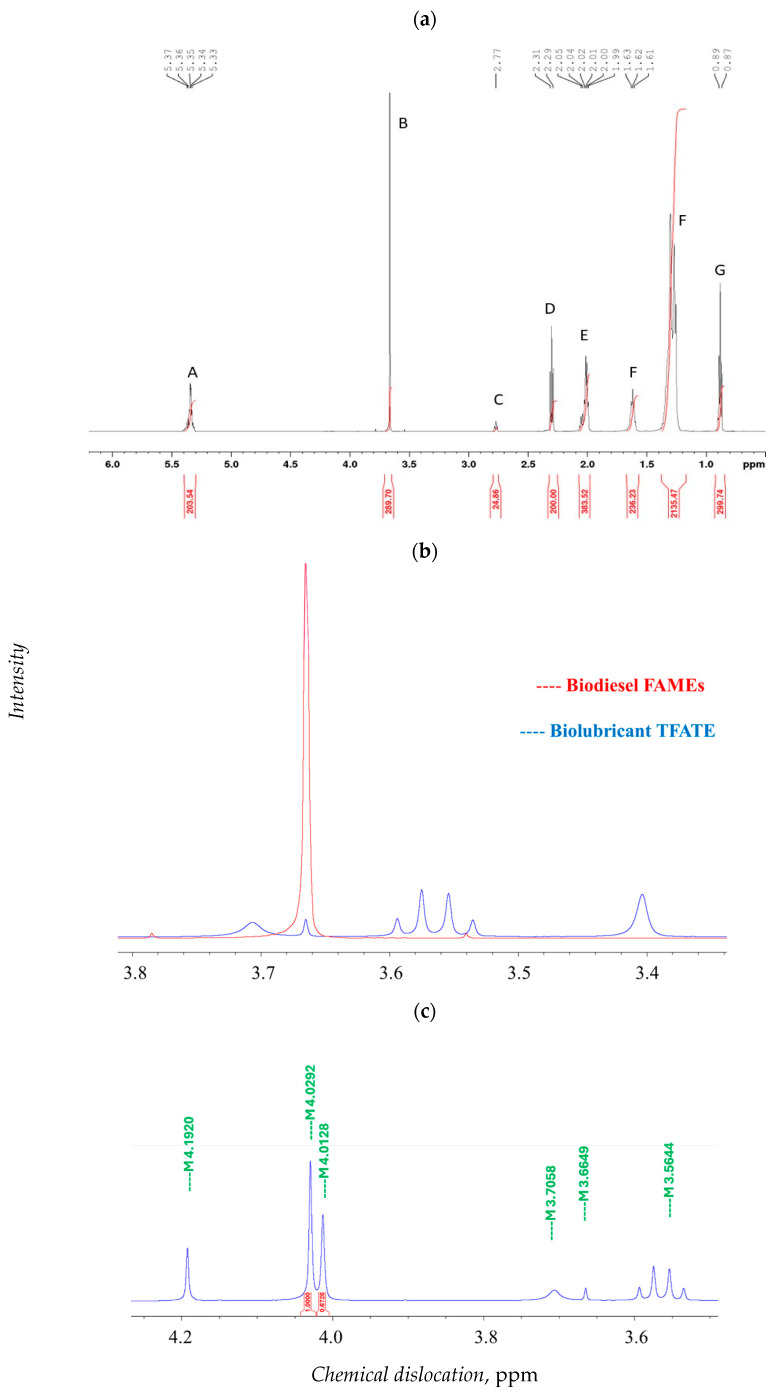
^1^H NMR analysis in CDCl3 (400 Mhz) for (**a**) safflower oil, (**b**) overlay of 1H NMR spectra of FAMEs and TFATE and (**c**) TFATE. Explanation of symbols A–G is given in the Supplementary Materials.

Finally, the conversion of safflower oil into FAMEs and, in turn, into TAFE was confirmed by the Hydrogen Nuclear Magnetic Resonance (^1^H NMR) spectra, see Figure 9. First, Figure 9a and Table S1 show the spectrum and the main peak positions contained in safflower oil. A range of 0.85–2.3 ppm was related to the aliphatic chain of the fatty acids with the acid proton CH_2_C=O located at 2.3 ppm [60]. The -CH=CH- proton signal was located at the 5.35 ppm position, indicating unsaturated fatty acids [60].

Figure 9b reports the spectra of the peak located at 3.66, characteristic of FAMEs, which diminished in the TFATE spectrum confirming the conversion, although with some residual FAME molecules. Figure 9c indicates the presence of signals around 3.6 and 4.8 ppm, which was due to the outcome of hydrogen bonds to unreacted hydroxyls that support the synthesis of mono- and di-substituted trimethyl propanol esters. In this case, the formation of tri- and di-esters of trimethyl propanol with dislocations at 4.01 and 4.03 ppm, respectively, was observed. The -CH_2_-O- proton of alcohol that results from the replacement of OH of TMP by the fatty acid chain of the methyl derivative was located at 4.19 ppm [60]. The diesters of TMP are reaction intermediates and, using the methodology of Nie et al. [62], it was possible to quantify TFATE conversion, the results of which were 31% for this study. As a reference, Aziz et al. [63] obtained 66.6% TFATE conversion using microwave-assisted pyrolysis at 130 °C, indicating that this conversion depended on the limiting reagent (i.e., TMP).

## 3. Materials and Methods

### 3.1. Synthesis of Heterogeneous Catalysts Using Avocado-Seed Char and Calcined Eggshells

**Figure 10:**
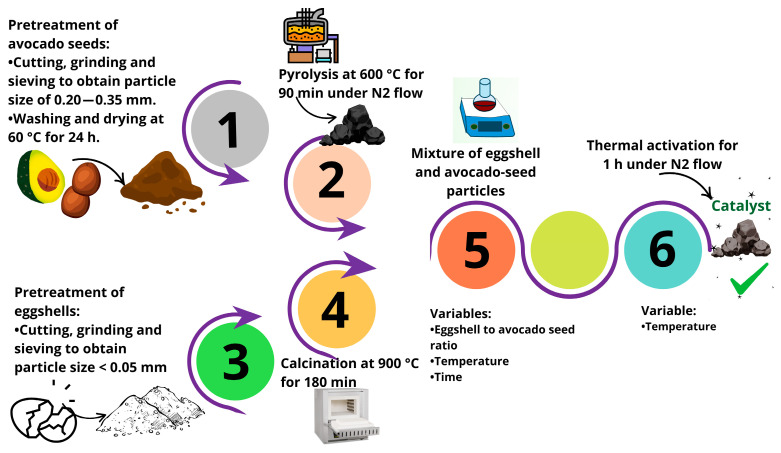
Experimental protocol for the preparation of heterogeneous catalysts from eggshell and avocado-seed char to produce biolubricant via transesterification.

The avocado seeds were cleaned with deionized water, cut into small pieces, and dried under ambient conditions for 2 days. They were then ground and sieved to obtain a particle size of 0.20–0.35 mm. Final cleaning using deionized water was performed on these particles to remove impurities, and they were dried at 60 °C for 24 h before pyrolysis. Avocado-seed particles were pyrolyzed at 600 °C for 90 min under a N_2_ flow rate of 400 mL/min using a Carbolite tubular furnace. The avocado-based char was cleaned with deionized water and dried for use as a catalyst support. On the other hand, clean eggshells were ground to powder and calcined at 900 °C in a muffle furnace for 180 min. The calcined eggshells and char were mixed at a specific eggshell/char ratio with 100 mL of deionized water. The suspension was stirred at a specific temperature for a specific amount of time, and the mixture was dried at 100 °C. The dried solid was subjected to thermal activation for 1 h in a tubular furnace under N_2_ flow to obtain the heterogeneous catalyst. Several samples of catalysts were prepared using this preparation route, where a Taguchi L9 experimental design was applied, see Table 1. The variables tested in the catalyst synthesis were temperature (40, 60, and 80 °C) and time (1, 2, and 3 h) for the mixing of char and calcined eggshells in the aqueous solution; eggshell/char ratio (0.5, 1, and 1.5 g/g); and temperature (700, 800, and 900 °C) for the activation of the eggshell-/char-based catalyst. Each sample obtained from this experimental design was used to produce the biolubricant. Figure 10 illustrates the protocol used for the synthesis of biomass-based heterogeneous catalysts.

### 3.2. Reactive Systems to Obtain FAMEs and TFATE

The production of TFATE was carried out using FAMEs as a reagent. First, the transesterification of commercial safflower oil (glycerides) was performed to obtain FAMEs using a methanol/oil molar ratio of 15/1 and 1% KOH (homogeneous catalyst) under continuous stirring at 60 °C for 5 h [64,65]. The upper phase (FAMEs + unreacted methanol) obtained from this reaction was separated from the lower phase (containing glycerin + unreacted oil) by decantation, and the methanol was removed by evaporation. FAMEs were washed with 20 mL of HNO_3_ (3% by volume) and stirred for 90 min. HNO_3_ solution was then separated from the FAMEs by decantation. The fatty acid profile and FAME content from this reactive system were quantified according to European Standard EN 14103 [66] using gas chromatography (GC), see the Supplementary Materials. In the second reaction, the biolubricant TFATE was synthesized from FAMEs using the alcohol trimethylolpropane (TMP) and the corresponding catalyst sample. The reaction conditions were as follows: 130 °C, 400 mbar, FAME/TMP molar ratio of 3/1, and 7.5% (by mass) catalyst. Once the second reaction was completed to obtain the biolubricant, the mixture was filtered to separate the heterogeneous catalyst. The filtered sample was cooled to room temperature, and the alcohol was separated by centrifugation. A sample was taken from the clarified phase of the centrifuged solution to quantify the remaining FAMEs using GC. TFATE yield (%) was calculated from the FAME material balance as follows:

(1) $$\%TFATE=\left(\frac{F_{O}-F_{t}}{F_{O}}\right)100$$

where *F_O_* is the amount of FAMEs before the biolubricant chemical reaction; and *F_t_* is the quantity of FAMEs remaining after the reaction. As stated, both quantities were quantified using GC. Three replicates (*n_r_* = 3) were performed to evaluate the reproducibility of all reactions, where an experimental error of <5% was achieved, and the mean values were reported.

The best conditions to prepare the eggshell-/char-based catalyst with the aim of improving biolubricant production were determined via the S/N ratio analysis [33], where the next expression was applied to calculate the corresponding experimental output:

(2) $$\frac{S}{N}=(-10)\log\left(\frac{1}{n_{r}}\sum_{i=1}^{n_{r}}\frac{1}{{\%TFATE}_{i}^{2}}\right)$$

Analysis of variance (ANOVA) of the Taguchi experimental design was carried out using the equations given in the Supplementary Materials.

### 3.3. Analysis of Transesterification Conditions to Obtain TFATE

The reaction kinetics to obtain TFATE were quantified using the best eggshell-/char-based catalyst at 120 and 150 °C with reaction times of 0.5–7 h. The reaction conditions were as follows: 400 mbar, FAME/TMP molar ratio of 3/1, and 5% (by mass) of the catalyst with respect to FAMEs [9]. The objective of these experiments was to determine the order and rate constant of this reactive system using the models given by Equations (3) and (4) [35]:

(3) $$Ln\frac{\left[TMP_{O}\right]}{\left[TMP\right]}=k_{1}t$$

(4) $$\frac{1}{\left[TMP\right]}-\frac{1}{\left[TMP_{O}\right]}=k_{2}t$$

where [*TMP_O_*] is the initial TMP concentration; [*TMP*] is the final TMP concentration at time *t* (both expressed in mmol/L); *t* is the reaction time (min); *k*_1_ (min^−1^) and *k*_2_ (L/mmol·min) correspond to the first- and second-order rate constants, respectively. The calculated reaction rate constants for the tested temperatures were used to calculate the activation energy (Ea, J/mol) of the biolubricant reactive system using the Arrhenius equation, see the Supplementary Materials.

An additional experimental set was applied to improve the biolubricant reaction conditions. The nominal conditions of this reactive system were initially set at a reaction temperature of 130 °C, FAME/TMP molar ratio of 3/1, 400 mbar, and catalyst concentration of 5% (by mass). The reaction conditions were then tested, and the remaining parameters were fixed at given nominal values: FAME/TMP molar ratio (2/1, 2.5/1, 3/1, and 3.5/1), reaction temperature (120, 130, 140, and 150 °C), and catalyst concentration (0.8, 1.6, 2.5, 5, 7.5, and 10% by mass).

### 3.4. Catalyst Reuse in TFATE Production

The analysis of eggshell-/char-based catalyst reuse was performed with a FAME/TMP molar ratio of 2/1, catalyst concentration of 5% (by mass), and reaction temperature of 150 °C. At the end of each reaction, the catalyst was separated from the biolubricant by filtration and cleaned with methanol at 40 °C for 30 min. This process was repeated four times, and the washed methanol was removed and replaced with pure methanol. Once the methanol-based washing was completed, the catalyst was dried at 100 °C and its reactivation was carried out at 900 °C for 1 h under a N_2_ flow rate of 400 mL/min. The reactivated catalyst was utilized to perform a new reaction under the conditions described, and this procedure was repeated 5 times.

A catalyst leaching test was also performed, while the Ca contents in the biolubricant and catalyst were quantified. For the leaching test, the catalyst was washed with 40 mL of methanol for three consecutive cycles, the alcohol was evaporated, and the resulting sample was diluted in HNO_3_ 10 % (by volume) to preserve the samples. Acidic demineralization of the biolubricant sample of each reaction cycle and the raw catalyst (before the first reaction) was carried out using concentrated HNO_3_, and the samples were diluted with deionized water for Ca quantification. The calcium content of these samples was determined using inductively coupled plasma optical emission spectroscopy, see details in the Supplementary Materials.

### 3.5. Characterization of Avocado Char, Eggshells, Catalysts, and Reaction Products

The biomass of avocado seeds was characterized using the Soxhlet extraction technique with water. This process was performed in triplicate at 100 °C for 6 h, and the quantity of solids contained in the resulting liquid and the mass loss after extraction were measured. The glucose concentration in the resulting liquid was determined using the phenolic–sulfuric acid method, while the NREL TP-510-42618 [67] method was applied to quantify the structural carbohydrate and lignin contents. TGA analysis of avocado biomass was also performed at 30–950 °C under a N_2_ atmosphere. Elemental analysis of the avocado seeds and char was carried out, where the samples were first dried at 100 °C for 24 h and then analyzed. FTIR was utilized to characterize the avocado biomass, char, reaction products, and catalysts, while ^1^H NMR was applied to characterize FAMEs and the biolubricant. The crystalline phases of selected catalyst samples were determined by XRD. Catalyst micrographs and elemental composition were obtained using SEM and EDS, respectively. BET surface area was determined by N_2_ physisorption. Details of all characterization techniques are reported in the Supplementary Materials.

## 4. Conclusions

This study introduced a low-cost and effective route to prepare a novel composite derived from chicken eggshells and avocado seeds that can be applied as a heterogeneous catalyst to produce 90% of biolubricant TFATE under optimized reaction conditions. The statistical analysis of catalyst preparation conditions revealed that the activation temperature of this composite had the highest impact on its catalytic performance in the tested reactive system. Transesterification kinetics, carried out at 120 and 150 °C, showed an endothermic behavior with calculated kinetic rate constants of 7.45 × 10^−3^–10.31 × 10^−3^ L/mmol·min and an activation energy of 15 kJ/mol to produce TFATE. Catalyst characterization demonstrated the important role of crystallinity and homogeneous loading of CaO on the avocado char surface to favor TFATE yield. Reuse tests showed that this novel catalyst can be used in five cycles, showing low calcium leaching where the catalyst deactivation was caused mainly by active-site poisoning. Therefore, further studies are required to identify an improved approach to reactivate the catalyst properties with the aim of expanding its lifetime. This novel biomass-based catalyst can be an alternative to help in the transition to replacing petrochemical lubricants with green and sustainable biolubricants.

## Figures and Tables

**Table 1:** Conditions tested for the preparation of heterogeneous catalysts from avocado-seed char and eggshell to produce the biolubricant (TFATE).

Catalyst Sample	Impregnation Temperature, °C	Impregnation Time, h	Ratio of Calcined Eggshell/Char, g/g	Activation Temperature, °C	TFATE, %
C1	40	1	0.5	700	49.75
C2	40	2	1	800	69.56
C3	40	3	1.5	900	60.46
C4	60	1	1	900	66.96
C5	60	2	1.5	700	59.18
C6	60	3	0.5	800	60.86
C7	80	1	1.5	800	47.66
C8	80	2	0.5	900	63.22
C9	80	3	1	700	47.06

**Table 2:** Statistical analysis of Taguchi L9 experimental design to prepare the heterogeneous catalysts from avocado seed char and eggshell to produce the biolubricant TFATE.

Variable Used to Prepare the Catalysts	Sum of Squares	Variance	Impact Order
Impregnation temperature, °C	3.87	1.94	2
Impregnation time, h	3.55	1.77	3
Ratio of calcined eggshell/char	0.74	0.37	4
Activation temperature, °C	4.73	2.36	1
Total	12.89		

**Table 3:** Calculated kinetic parameters for FAME transesterification to produce the biolubricant (TFATE).

	First-Order Rate Constant, min^−1^		Second-Order Rate Constant, L/mmol·min	
Parameter	120 °C	150 °C	120 °C	150 °C
k	3.74 × 10^−3^	3.88 × 10^−3^	7.39 × 10^−3^	10.25 × 10^−3^
R^2^	0.954	0.937	0.968	0.977

**Table 4:** Results for TFATE production using different heterogeneous catalysts.

Source of FAMEs	Alcohol	Catalyst	Molar Ratio of FAME–Alcohol	Reaction Conditions	Biolubricant Formation, %	Reference
Palm oil	TMP	Mixed oxides of CaO and SrO	6:1	180 °C, 0.002 atm, 1% (by mass), 4 h	88	[38]
Waste cooking oil	TMP	Calcined waste cockle shell	3:1	130 °C, 4% (by mass), 4 h	97	[21]
Neem seed oil	TMP	Calcined eggshells subsequently activated with H_3_PO_4_	2:1	100 °C, 4.5% (by mass), 4 h	---	[25]
Methyl oleate	TMP	K_2_CO_3_/activated carbon	4:1	120 °C, 20% (by mass), 4 h	94	[20]
Safflower oil	TMP	CaO from eggshell supported on avocado-seed-derived char	2:1	150 °C, 0.4 atm, 5% (by mass), 5 h	90	This study

**Table 5:** Results of eggshell-/char-based catalyst reuse and calcium leaching into TFATE.

Reaction Cycle	TFATE Formation, %	Ca Content in TFATE, mg/g
0	90.02	0.062
1	84.43	0.092
2	73.21	0.053
3	69.76	0.048
4	57.00	0.039
5	48.21	0.038

**Table 6:** Elemental analysis of avocado seed and its char.

Element, %	Avocado Seed	Avocado Char
Carbon	44.25	84.04
Hydrogen	6.59	2.23
Nitrogen	0.53	1.61
Sulfur	0.09	0.04
Oxygen	48.54	12.08

**Table 7:** Elemental analysis of selected eggshell-/char-based catalysts used in TFATE production.

	Sample		
Element, %	Best Catalyst	Intermediate Catalyst C5	Worst Catalyst C9
Carbon	20.3	45.8	45.0
Oxygen	35.2	28.2	29.3
Calcium	42.8	25.2	24.9
Others	0.8	0.8	0.8

**Table 8:** Composition of fatty acids of FAMEs determined from GC analysis.

Fatty Acid	Composition, %
Myristic (C14:0)	0.05
Palmitic (C16:0)	4.66
Stearic (C18:0)	0.38
Oleic (C18:1)	90.84
Linoleic (C18:2)	3.27
Arachidonic (C20:4)	0.79

## Data Availability

The original contributions presented in this study are included in the article/Supplementary Materials. Further inquiries can be directed to the corresponding author.

## Supplementary Materials

The following supporting information can be downloaded at https://www.mdpi.com/article/10.3390/molecules30214280/s1. Section S1: FAME quantification; Section S2: Analysis of variance of Taguchi experimental design; Section S3: Analytical equipment and procedures; Section S4: Calculation of activation energy; Figure S1: Thermogravimetric analysis of avocado-seed biomass; Figure S2: EDS calcium mapping of selected samples of eggshell-/char-based catalysts used for biolubricant TFATE formation; Table S1: Assignment of ^1^H NMR peaks for safflower oil.

## Abbreviations

The following abbreviations are used in this manuscript:

FAME	Fatty acid methyl ester
TFATE	Trimethylolpropane fatty acid triester
TMP	Trimethylolpropane
