# Supplementary material for: A Novel Biomass-Based Catalyst Composite Using Waste Chicken Eggshells and Avocado Seeds for Biolubricant Production: Synthesis Route, Catalytic Property Characterization, and Performance
Source: Molecules. 2025 Nov 3;30(21):4280. doi: 10.3390/molecules30214280 (PMC12609544; doi:10.3390/molecules30214280)
Supplement: Supplementary file 1 [file molecules-30-04280-s001.zip › molecules-3847993-supplementary.pdf]

SUPPLEMENTARY INFORMATION

S1. FAME quantification

Thermo Scientific Trace 1300 gas chromatography equipment with a flame ionization detector was used in this study to quantify FAME. TG-5 SILMS column of 30 m x 0.25 mm x 0.25 µm composed of 5% diphenyl and 95% dimethylarylenesiloxane was used. The analysis method consisted of a splitless injection temperature of 250 °C, using a flow of helium (2.5 mL) in the column. The detector temperature was set to 300 °C. The temperature program consisted of 40 °C for 2.5 min, followed by a temperature increase at a rate of 10 °C/min until it reached 280 °C, and was finally maintained at 280 °C for 10 min. FAME formation (%) was determined using Equation (S1) where heptadecanoate was the internal standard.

$$\%FAME = \left(\frac{\sum A_T - A_{std}}{A_{std}}\right) * \left(\frac{C_{std} * V_S}{m}\right) * 100 \quad \text{(S1)}$$

where ΣA_T is the sum of the area of the internal standard and the area of peaks corresponding to the FAME, *m* is the mass of the sample in g; A_{std}, C_{std} (mg/mL), and V_S (mL) are the area, concentration and volume of the internal standard, respectively.

S2. Analysis of variance of Taguchi experimental design

The analysis of variance (ANOVA) was carried out to know the statistical influence of the synthesis variables on the response variable. Specifically, Equation S2 was used to calculate the total sum of squares (SS_T) and equations S3 and S4 for the sum of squares and variance for the factor F (SS_F and σ_F), respectively:

$$SS_T = \sum_{i=1}^{n_T} y_i^2 - \frac{T_t^2}{n_T} \quad \text{(S2)}$$

$$SS_F = \sum_{i=1}^{k_F} \frac{F_i^2}{n_{F_i}} - \frac{T_t^2}{n_T} \quad \text{(S3)}$$

$$\sigma_F = \frac{SS_F}{GL_F} \quad \text{(S4)}$$

where n_T corresponds to the number of experimental data, y_i represents the value obtained with the Signal/Noise ratio, T_t is the sum of the response variable in all the experimental runs, k_F denotes the number of levels of the factor *F*, F_i is the sum of the response variable of the experimental runs at level *i* of the factor *F*, n_{Fi} indicates the number of experimental runs at level *i* of the factor *F* and GL_F are the degrees of freedom of the factor *F*, which is found by subtracting 1 from k_F.

S3. Analytical equipment and procedures

Fourier transform infrared spectroscopy (FTIR) was used to characterize the reagents, reaction products, and catalysts with a Thermo Scientific Nicolet iS10 infrared spectrophotometer, where KBr pellets were used to dilute the samples that were analyzed in a wave number range of 4000-400 cm^{-1} with 32 scans. Hydrogen nuclear magnetic resonance (HNMR) was performed on both FAME and the biolubricant using a Bruker BioSpin 900 MHz AVANCE series NMR spectrometer. The crystalline phases of the catalysts were determined by X-ray diffraction (XRD) using an Empyrean Malvern-Panalytical diffractometer equipped with a PIXel ID detector. The analysis was performed in a range of 10 to 110 °2θ with a step size of 0.026 °, a CuKα1 radiation of 1.5405 Å and with a generator configuration of 40 mA and 45 kV. Finally, phase identification was performed using HighScore Plus software and the PDF-2 database. Micrographs and elemental composition of the catalysts were obtained with a TESCAN VEGA 3 LMU 15 kV equipment with a resolution of 3 nm that incorporates a Bruker EDS-type chemical analysis system and an 80 mm² SDD-type detector. Thermo Scientific ICAP 7000 series inductively coupled plasma optical emission spectrometer (ICP-OES) was used for calcium quantification with a calibration curve in the range of 0.01 – 10 mg/L. BET surface area of catalyst samples was determined via N_2 physisorption.

S4. Calculation of activation energy

The reaction activation energy (Ea, J/mol) was calculated using the Arrhenius equation

$$k = Ae^{-\frac{E_a}{RT}} \qquad \text{(S5)}$$

where A is the frequency factor, R is the ideal gas constant (8.314 J/mol · K), and T is the reaction temperature (K).

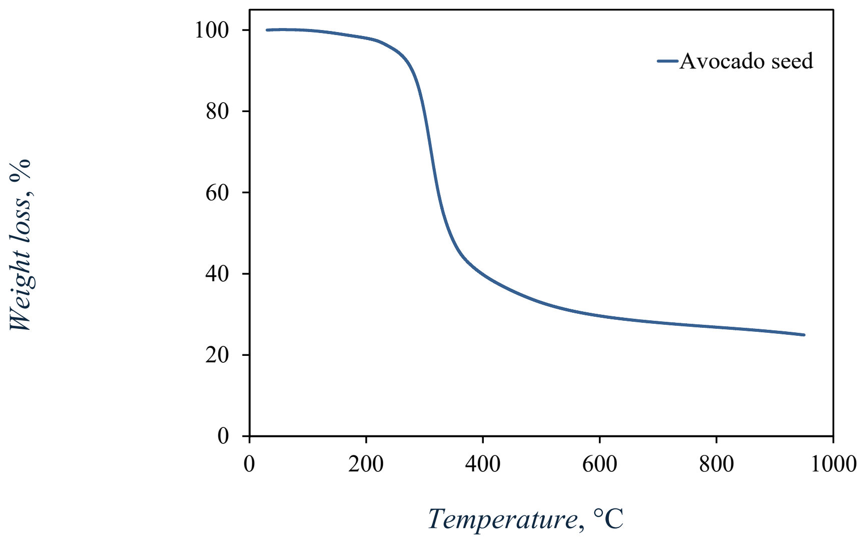

Figure S1. Thermogravimetric analysis of avocado seed biomass.

a) Best catalyst b) Intermediate catalyst C5 c) Worst catalyst C9

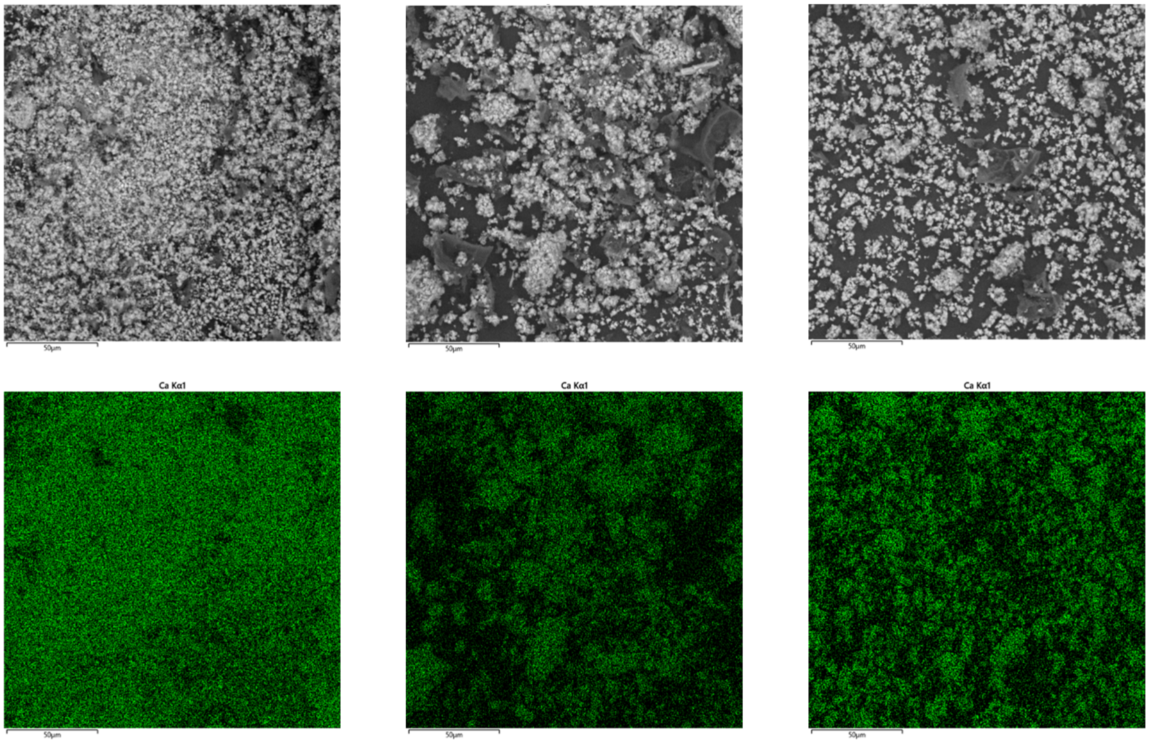

Figure S2. EDS calcium mapping of selected samples of eggshell/char-based catalysts used for biolubricant TFATE formation.

Table S1. Assignment of HNMR peaks for safflower oil.

Peak	*Identified hydrogens*
A	Methylene -CH=CH-
B	Methyl ester $–CH_3$
C	$–CH_2–$ between two non-conjugated double bonds
D	$–CH_2$ adjacent to carbonyl group (C=O)
E	$–CH_2–$ adjacent to carbonyl group (C=O)
F	$–CH_2–$ of aliphatic
G	Ending of the aliphatic chain $–CH_3$
